# (−)-Guaiol regulates RAD51 stability via autophagy to induce cell apoptosis in non-small cell lung cancer

**DOI:** 10.18632/oncotarget.11540

**Published:** 2016-08-23

**Authors:** Qingyuan Yang, Jianchun Wu, Yingbin Luo, Nan Huang, Ni Zhen, Yun Zhou, Fenyong Sun, Zhi Li, Qiuhui Pan, Yan Li

**Affiliations:** ^1^ Department of Clinical Laboratory Medicine, Tenth People's Hospital of Tongji University, Shanghai, 200072, China; ^2^ Department of Oncology, Shanghai Municipal Hospital of Traditional Chinese Medicine, Shanghai University of Traditional Chinese Medicine, Shanghai, 200071, China; ^3^ Department of Clinical Laboratory Medicine, Yangpu Hospital of Tongji University, Shanghai, 200090, China; ^4^ Central Laboratory, Tenth People's Hospital of Tongji University, Shanghai, 200072, China

**Keywords:** (−)-Guaiol, RAD51, autophagy, chemosensitivity, DSBs

## Abstract

(−)-Guaiol, generally known as an antibacterial compound, has been found in many medicinal plants. Its roles in tumor suppression are still under investigation. In the study, we mainly focused on exploring its applications in dealing with non-small cell lung cancer (NSCLC) and the underlying mechanisms. Here, we show that (−)-Guaiol significantly inhibits cell growth of NSCLC cells both *in vitro* and *in vivo*. Further high throughput analysis reveals that RAD51, a pivotal factor in homologous recombination repair, is a potential target for it. The following mechanism studies show that (−)-Guaiol is involved in cell autophagy to regulate the expression of RAD51, leading to double-strand breaks triggered cell apoptosis. Moreover, targeting RAD51, which is highly overexpressed in the lung adenocarcinoma tissues, can significantly increase the chemosensitivity of NSCLC cells to (−)-Guaiol both *in vitro* and *in vivo*. All in all, our studies provide an attractive insight in applying (−)-Guaiol into NSCLC treatments and further suggest that knockdown of oncogenic RAD51 will greatly enhance the chemosensitivity of patients with NSCLC.

## INTRODUCTION

Non-small cell lung cancer (NSCLC) accounts for nearly 80-85% of total lung cancer [1], which is further divided into adenocarcinoma, squamous carcinoma and large-cell carcinoma based on the pathology [2]. The five year survival rates following lung resection for NSCLC are I A (73%), I B (54%), II A (48%), II B (38%), III A (25%), respectively [3]. NSCLC presents a heavy burden for countries worldwidely, for that it grows very fast and is often diagnosed in advanced stage [4]. Currently, patients with advanced-stage NSCLC are mostly treated with chemotherapeutic drugs, such as platinum agents (cisplatin, carboplatin), taxanes (paclitaxel, docetaxel), and oncogenetic mutation drivers-targeted agents (Apatinib, erlotinib, gefitinib, crizotinib) [5, 6]. Unfortunately, although the targeted therapeutic drugs have substantial impacts on cancer inhibition, the resistance of patients against these drugs usually develops within 1-2 years by activating compensatory signaling pathways or generating novel mutations [7]. Therefore, it is imperative to discover new drugs and to elucidate their potential mechanisms to help guide a more rational mechanism-based individual therapy for NSCLC patients.

(−)-Guaiol, the formula of which is C_15_H_26_O, is a key component of many medicinal plants, especially traditional Chinese medicines [8], and composes a large number of guaiane natural products [9, 10], which have been proved to possess pronounced antibacterial activities [11]. Recently, some researchers have found that the derivatives of guaiane-type sesquiterpenoids play an important role in tumor inhibition [12, 13]. However, the underlying mechanisms are still unclear.

In the study, we provided sufficient evidence to support that (−)-Guaiol was a potential antitumor candidate for NSCLC patients. And mechanistically, we discovered that (−)-Guaiol significantly targeted RAD51 for degradation, which was highly overexpressed in the lung adenocarcinoma tissues, by inducing cell autophagy, thus leading to the double strand breaks (DSBs)-triggered cell apoptosis in NSCLC cells. Besides, inhibition of RAD51 could greatly increase chemosensitivity of NSCLC cells to (−)-Guaiol. As a result, our data support that RAD51 is an oncogene in NSCLC tumorigenesis and it can serve as a potential target for (−)-Guaiol in the treatments of NSCLC patients. Alternatively, our results implicate that combination administration of specific RAD51 inhibitors and RAD51-targeted chemodrugs could help reduce the occurrence of acquired resistance, thus providing an attractive strategy for the treatments of NSCLC patients.

## RESULTS

### (−)-Guaiol inhibits cell growth, arrests cells in S phase and induces cell apoptosis in NSCLC cells both *in vitro* and *in vivo*

**Figure 1:**
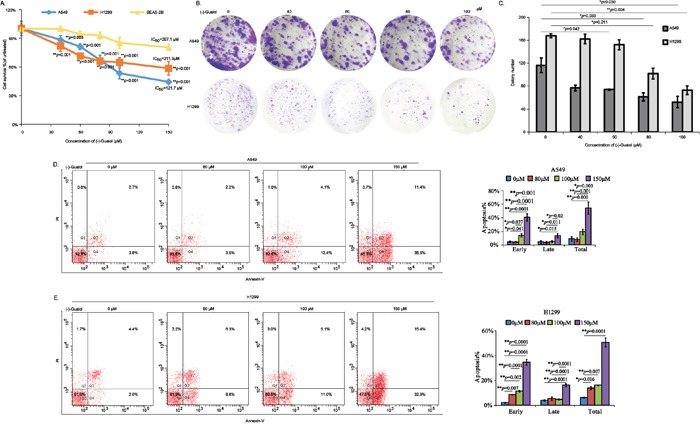
*In vitro* evaluation of antitumor activity of (−)-Guaiol in NSCLC cells **A.** Cell survival analysis of NSCLC cells (A549 and H1299) and normal lung cells BEAS-2B cells treated with different concentrations of (−)-Guaiol using CCK8 assays. Data from three independent experiments were represented as means ± STD. IC_50_ values were 121.7 μM for A549 cells, 211.5 μM for H1299 cells, and 297.1 μM for BEAS-2B cells, respectively. **B-C.** Colony formation assays of inhibition roles of (−)-Guaiol on A549 cells and H1299 cells (B). Colony numbers from three independent experiments were statistically analyzed using ANOVA tests (C). **D-E.** Flow cytometry assays of cell apoptosis of A549 (D) and H1299 (E) cells treated with indicated concentrations of (−)-Guaiol. Data from three independent experiments were represented as means ± STD and subjected to ANOVA tests, respectively.

Firstly, to investigate the potential antitumor activities of (−)-Guaiol in NSCLC development, we carried out the CCK8 assays using A549 cells and H1299 cells, taking normal lung cells BEAS-2B as a control. As demonstrated in Figure 1A, (−)-Guaiol dramatically inhibited cell growth of A549 cells and H1299 cells with IC_50_ values 121.7 μM and 211.5 μM, respectively. Whereas its IC_50_ value for BEAS-2B cells was 297.1 μM. Moreover, results from colony formation assays further confirmed that (−)-Guaiol suppressed the growth of A549 and H1299 cells (Figure 1B and 1C). Afterwards, the biological role of (−)-Guaiol in inducing cell apoptosis was explored using flow cytometry assays. As shown in Figure 1D and 1E, (−)-Guaiol significantly increased early apoptosis (early), late apoptosis (late) and total apoptosis (total) in a concentration dependent manner in both A549 and H1299 cells. Additionally, results from cell cycle analysis showed that (−)-Guaiol obviously arrested cells in S phase in both A549 and H1299 cells (Supplementary Figure S1). Above all, the *in vitro* assays support that (−)-Guaiol is a novel antitumor agent.

**Figure 2:**
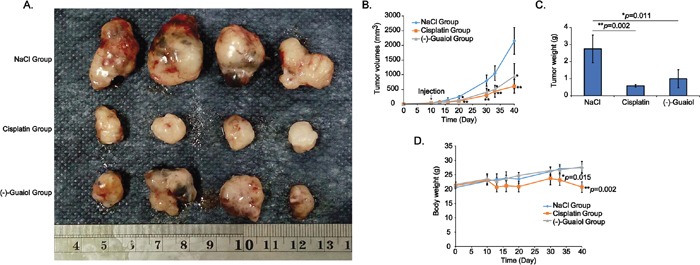
*In vivo* evaluation of tumor inhibition activity of (−)-Guaiol on NSCLC cells using the nude mice xenograft tumor model **A.** Tumors dissected from tumor-bearing nude mice intraperitoneally injected with NaCl, cisplatin or (−)-Guaiol. **B.** Statistical analysis of tumor volumes (mm^3^) during the chemotherapeutic experiment. **C.** Statistical analysis of tumor weights (g) at the terminal time point. Error bars, STD. **D.** Statistical analysis of body weights (g) of nude mice treated with or without (−)-Guaiol at the end of the experiment. Data were represented as means ± STD.

Besides, results from *in vivo* tumor chemotherapy assays showed that the volumes and weights of tumors from (−)-Guaiol treated group were statistically smaller and less than those from NaCl treated group, similar to the cisplatin treated group (Figure 2A-2C). Intriguingly, the body weights of nude mice from cisplatin treated group were more greatly reduced than those of (−)-Guaiol treated group, in comparison with NaCl treated group (Figure 2D), implying that (−)-Guaiol might be safer than cisplatin for NSCLC patients. It is noteworthy that (−)-Guaiol has no toxicity to the kidney and liver of these nude mice (data not shown).

### High throughput identification of differentially expressed genes in (−)-Guaiol treated A549 cells

**Figure 3:**
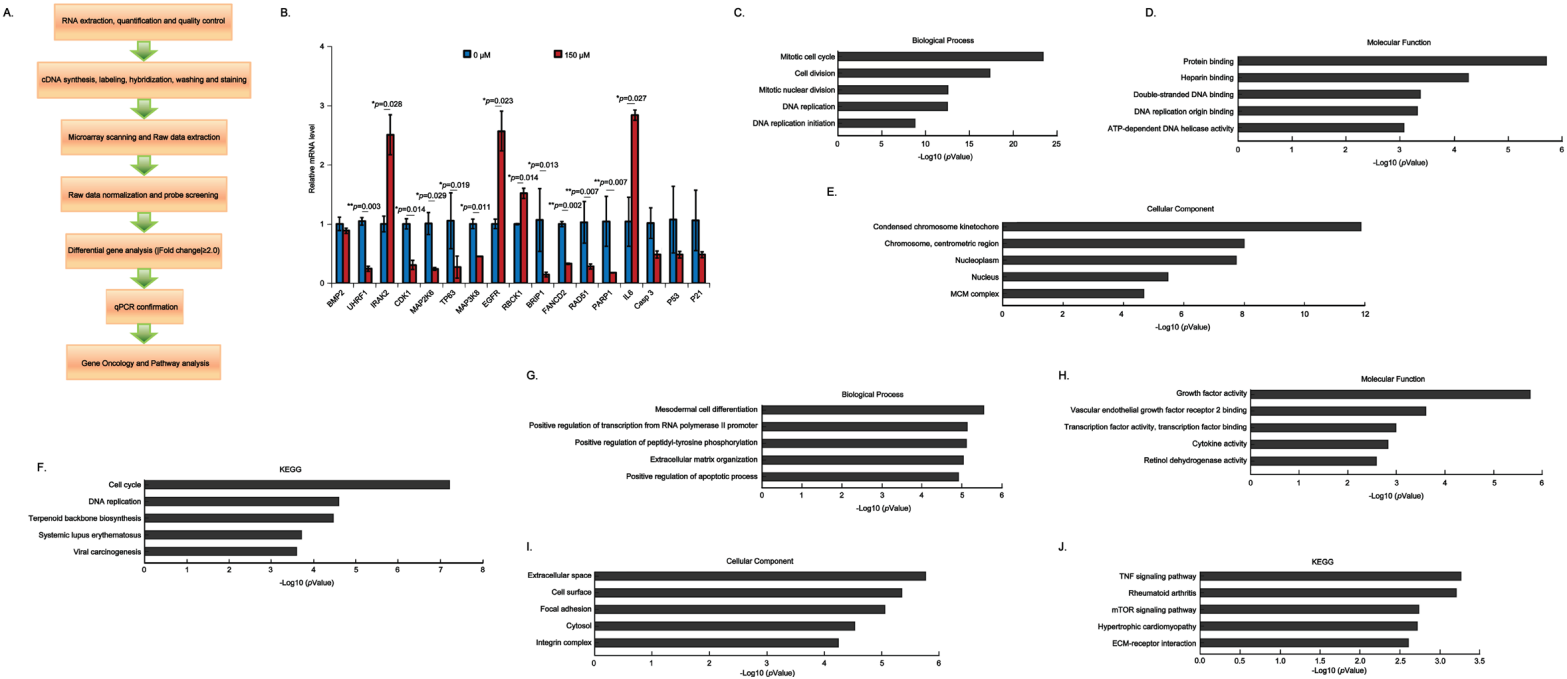
High throughput investigation of the differentially expressed genes in (−)-Guaiol treated NSCLC cells **A.** The experimental steps for the high throughput analysis using the Affymetrix Human PrimeView microarray. **B.** Selective confirmation of results from high throughput analysis usingthe quantitative PCR (qPCR) assays. Data were represented as means ± STD and statistically analyzed using the Student's *t*-test. **C-F.** The biological process analysis (C), molecular function analysis (D), cellular component analysis (E) and KEGG analysis (F) of downregulated genes identified from the above microarray. The top five categories were shown. **G-J.** The biological process analysis (G), molecular function analysis (H), cellular component analysis (I) and KEGG analysis (J) of upregulated genes identified from the above microarray. The top five categories were shown.

In order to identify the potential targeted genes of (−)-Guaiol in NSCLC cells, we employed the high throughput analysis using Affymetrix Human PrimeView microarray. The experimental steps were shown in Figure 3A. Based on the criteria that the fold change ≥2.0, we finally identified 474 upregulated genes and 391 downregulated genes (data now shown). Afterwards, we selected 4 upregulated genes (IRAK2, EGFR, RBCK1, IL6), 9 downregulated genes (UHRF1, CDK1, MAP2K6, TP63, MAP3K8, BRIP1, FACN2, RAD51, PARP1) and 4 genes (BMP2, Caspase 3, P53, P21), which are well-known in participation in apoptosis but not identified in this work, to be confirmed using quantitative PCR (qPCR) assays. Consistently, the statistical analysis revealed that UHRF1 (**p=0.003), CDK1 (*p=0.014), MAP2K6 (*p=0.029), TP63 (*p=0.019), MAP3K8 (*p=0.011), BRIP1 (*p=0.013), FANCD2 (**p=0.002), RAD51 (**p=0.007), PARP1 (**p=0.007) were significantly downregulated in (−)-Guaiol treated A549 cells, in comparison with untreated cells. Moreover, IRAK2 (*p=0.028), EGFR (*p=0.023), RBCK1 (*p=0.014) and IL6 (*p=0.027) were also dramatically upregulated whereas BMP2, Caspase 3 (Casp 3), P53 and P21 showed no statistical significance (Figure 3B).

Subsequently, using the online GO analysis database (http://amigo.geneontology.org/rte), we further analyzed the biological process, molecular function, cellular component and KEGG signaling pathways of those downregulated and upregulated genes. Interestingly, the downregulated genes were mostly involved in mitotic cell cycle, cell division, mitotic nuclear division and DNA replication in biological process category (Figure 3C), functioned in protein binding, heparin binding, DSB binding, DNA replication origin binding and ATP-dependent DNA helicase activity in molecular function category (Figure 3D), composed of condensed chromosome kinetochore, chromosome centrometric region, nucleoplasm, nucleus and MCM complex in cellular component category (Figure 3E), participated in cell cycle, DNA replication, terpenoid backbone biosynthesis, systematic lupus erythematosus and viral carcinogenesis in KEGG category (Figure 3F). On the other hand, those upregulated genes were mainly participated in mesodermal cell differentiation, positive regulation of transcription from RNA polymerase II promoter, positive regulation of peptidyl-tyrosine phosphorylation, extracellular matrix organization, positive regulation of apoptotic process in biological process category (Figure 3G), involved in growth factor activity, vascular endothelial growth factor receptor 2 binding, transcription factor activity and binding, cytokine activity and retinol dehydrogenase activity in molecular function category (Figure 3H), existed in extracellular space, cell surface, focal adhesion, cytosol and integrin complex in cellular component (Figure 3I), functioned in TNF signaling pathway, rheumatoid arthritis, mTOR signaling pathway, hypertrophic cardiomyopathy, ECM-receptor interaction in KEGG category (Figure 3J). Whatever, these activities of downregualted and upregulated genes indirectly reflected the biological roles of (−)-Guaiol in NSCLC cells.

### (−)-Guaiol targeted RAD51 to generate DSBs in NSCLC cells

**Figure 4:**
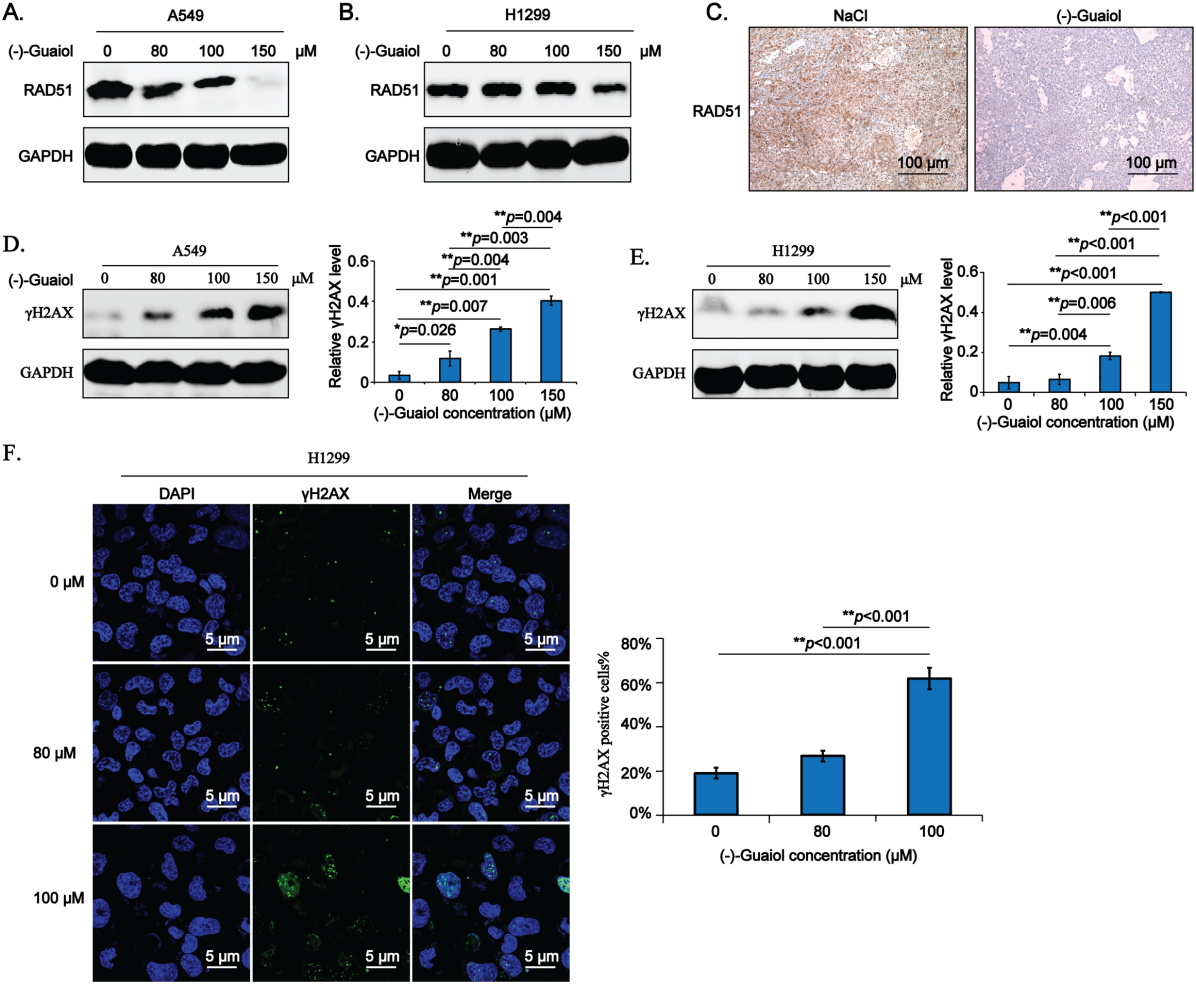
(−)-Guaiol is a DNA damaging agent by targeting RAD51 in NSCLC cells **A-B.** Immunostaining of total protein extracted from (−)-Guaiol treated A549 (A) or H1299 (B) cells with indicated antibodies. **C.** Immunohistochemical (IHC) analysis of RAD51 in tumor tissues from NaCl or (−)-Guaiol treated nude mice. The representative data were shown. Scale bar, 100 μm. **D-E.** Immunostaining of total protein extracted from (−)-Guaiol treated A549 (D) and H1299 (E) cells to test γH2AX expression, taking GAPDH as the internal reference. The gray values of γH2AX bands against GAPDH bands (defined as relative γH2AX level) from three independent experiments were subjected to ANOVA test. **F.** Immunofluorescence (IF) analysis of foci formation of γH2AX in H1299 cells treated with indicated concentrations of (−)-Guaiol. Representative results were shown. The percentage of γH2AX positive cells in 200 cells (γH2AX positive cells%) were statistically analyzed using ANOVA test.

According to the data from above high throughput analysis, we mainly focused on studying RAD51, a key homologous recombination repair (HRR) factor involved in forming a nucleoprotein filament by polymerizing onto single-stranded DNA at the processed DNA breaks via its strand transferase activity [14], as a potential target of (−)-Guaiol in NSCLC cells. Firstly, we employed the immunostaining assays to evaluate the expression of RAD51 in (−)-Guaiol treated A549 and H1299 cells. As shown in Figure 4A and 4B, RAD51 was significantly downregulated in 150 μM (−)-Guaiol treated NSCLC cells. Additionally, data from immunohistochemical (IHC) assays showed that RAD51 was also dramatically reduced in (−)-Guaiol treated tumor tissues (Figure 4C).

Subsequently, we explored whether downregulation of RAD51 by (−)-Guaiol would lead to the accumulation of DSBs in NSCLC cells. Data from immunostaining assays showed that the DSB biomarker, γH2AX [15], was increased depending on the concentrations of (−)-Guaiol in both A549 (Figure 4D) and H1299 (Figure 4E) cells. Moreover, the formation of γH2AX foci tested by confocal immunofluorescenc (IF) assays demonstrated that the DSBs generated in (−)-Guaiol treated H1299 cells were also elevated in a concentration-dependent manner (Figure 4F). Taken above data into consideration, we think that (−)-Guaiol is a DNA-damaging agent by targeting RAD51.

### (−)-Guaiol is involved in the autophagy-lysosome pathway to regulate RAD51 expression in NSCLC cells

**Figure 5:**
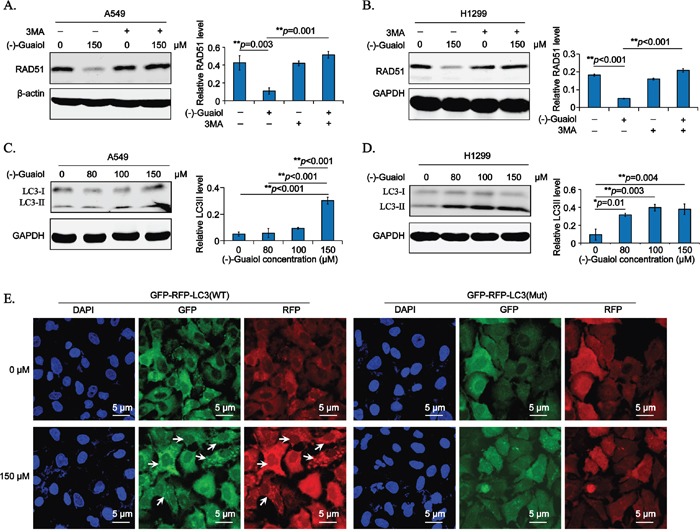
(−)-Guaiol regulates RAD51 expression via autophagy in NSCLC cells **A-B**. Immunostaining of total protein extracted from A549 (A) or H1299 (B) cells treated with or without (−)-Guaiol, followed by the exposure to 5 mM 3MA in serum free medium for 6 h, with indicated antibodies. The relative RAD51 levels from three independent experiments were statistically analyzed using ANOVA test. **C-D.** Immunostaining of total protein from A549 (C) or H1299 (D) cells treated with indicated concentrations of (−)-Guaiol with indicated antibodies. The relative LC3 II levels from three independent experiments were statistically analyzed using ANOVA test. **E.** Representative confocal IF data from autophagic flux assays of stable A549 cells infected with GFP-RFP-LC3 WT or Mut lentivirus and treated with or without (−)-Guaiol. The LC3 puncta in cytoplasm were indicated by the white arrow. Scale bar, 5 μm.

It is well-known that there are two main proteolytic systems in mammalian, one is the proteasome-ubiquitination system, the other is lysosomal system or autophagy [16]. Therefore, to clarify the underlying mechanisms of (−)-Guaiol in downregulating RAD51 in NSCLC cells, the autophagy inhibitor 3MA and proteasome inhibitor MG132 were put into practice, respectively. Surprisingly, results from immunostaining displayed that 3MA dramatically rescued the reduction of RAD51 in (−)-Guaiol treated A549 cells (Figure 5A) and H1299 cells (Figure 5B). However, RAD51 was still significantly downregulated in MG132 and (−)-Guaiol treated A549 cells (Supplementary Figure S2A) and H1299 cells (Supplementary Figure S2B). Therefore, our data support that (−)-Guaiol regulates the expression of RAD51 via lysosomal pathway.

Afterwards, to further confirm the role of (−)-Guaiol in autophagy, we detected the canonical autophagy biomarker, LC3 II [17], using immunostaining assays. As shown in Figure 5C and 5D, the relative LC3 II expression was apparently increased in both A549 and H1299 cells, especially in 150 μM (−)-Guaiol treated cells. Moreover, the dynamic autophagy flux assays were used to exclude the possibility that the increase of LC3 II was resulted from the inhibition of autophagy at the lysosome degradative stage. As shown in Figure 5E and Supplementary Figure S3, both of the GFP-LC3 (Green) and RFP-LC3 (Red) puncta in the cytoplasm were significantly increased in (−)-Guaiol treated A549 cells, whereas there were no visible LC3 puncta in GFP-RFP-LC3 (Mut) infected A549 cells. Above all, our data confirm that (−)-Guaiol plays an active role in inducing autophagy to reduce the expression of RAD51, leading to the accumulation of DSBs in NSCLC cells.

### (−)-Guaiol dramatically induces DSBs-triggered cell apoptosis in NSCLC cells

**Figure 6:**
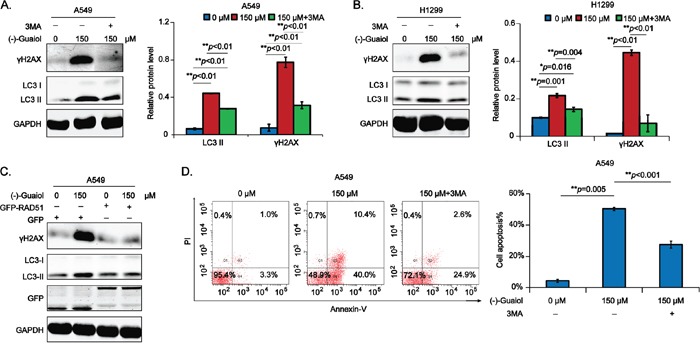
(−)-Guaiol obviously promotes DSB-triggered cell apoptosis in NSCLC cells via autophagy **A-B.** Immunostaining of total protein from A549 (A) or H1299 (B) cells treated with or without (−)-Guaiol, followed by the exposure to 3MA, with indicated antibodies. The relative γH2AX levels were statistically analyzed using ANOVA test. **C.** Immunostaining of total protein from A549 cells, transfected with GFP or GFP-RAD51 and treated with or without (−)-Guaiol, with indicated antibodies. **D.** Flow cytometry assays of cell apoptosis of A549 cells treated with or without (−)-Guaiol, followed by the exposure to 3MA. The percentage of total apoptotic cells (Cell apoptosis%) from three independent experiments were represented as means ± STD and statistically analyzed using ANOVA test.

Our above data had illustrated that (−)-Guaiol was a DNA-damaging agent by targeting RAD51 for degradation via autophagy. It has been widely recognized that the accumulation of DSBs in tumor cells will finally lead to cell apoptosis [18]. Therefore, we hypothesized that (−)-Guaiol induced DSBs-triggered cell apoptosis by targeting RAD51 for degradation via autophagy. To consolidate our speculation, we firstly examined whether 3MA could reverse the generation of γH2AX in (−)-Guaiol treated NSCLC cells. As shown in Figure 6A and 6B, 3MA dramatically inhibited the high expression of γH2AX induced in (−)-Guaiol treated A549 and H1299 cells. Next, data from immunostaining assays showed that both LC3 II and γH2AX were reduced in RAD51 overexpressed A549 cells treated with (−)-Guaiol (Figure 6C), indicating that (−)-Guaiol inhibited tumor growth by targeting RAD51 for lysosome degradation to induce DSBs-triggered cell apoptosis. Moreover, data from flow cytometry assays showed that the cell apoptosis in (−)-Guaiol treated cells was statistically inhibited by 3MA (Figure 6D). Surprisingly, using the pan-caspase inhibitor Z-VAD, the cell apoptosis in (−)-Guaiol treated A549 cells was similarly suppressed as 3MA (Supplementary Figure S4A and S4B), implicating that the RAD51 deficiency generated DSBs triggered cell apoptosis in (−)-Guaiol treated NSCLC cells by increasing caspase activities.

### Downregulation of RAD51 reduces the therapeutic resistance of NSCLC cells to (−)-Guaiol both *in vitro* and *in vivo*

**Figure 7:**
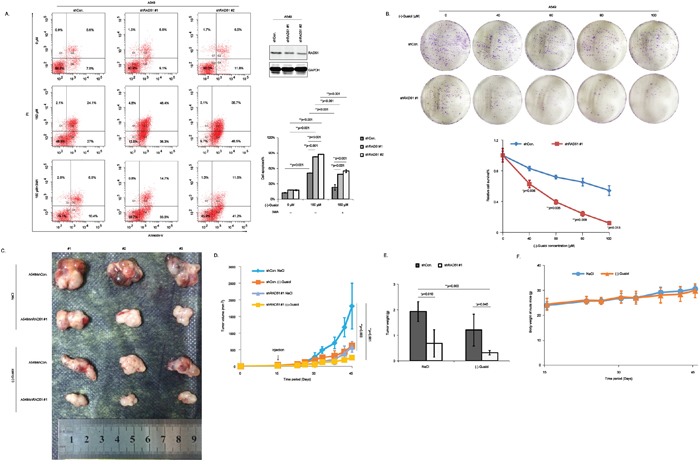
Knockdown of RAD51 significantly increases the sensitivity of NSCLC cells to (−)-Guaiol both *in vitro* and *in vivo* **A.** Cell apoptosis analysis of stable A549 cells, infected with shCon. or shRAD51 #1, #2 lentivirus and treated with or without (−)-Guaiol, followed by the exposure to 3MA, were analyzed using flow cytometry assays. Cell apoptosis% from three independent experiments were represented as means ± STD and further statistically analyzed using ANOVA test. Downregulation of RAD51 by shRNAs were confirmed using immunostaining assays. **B.** Clonogenic survival analysis of stable A549 cells, infected with shCon. or shRAD51 #1 and treated with indicated concentrations of (−)-Guaiol. The relative cell survival% was statistically analyzed using Student's *t*-test. **C-F.** *In vivo* chemosensitivity analysis of A549/shCon. and A549/shRAD51 #1 using the tumor-bearing nude mice. The tumors dissected from nude mice were shown (C). The tumor volumes (mm^3^) during the experiment were recorded and statistically analyzed (D). The tumor weights (g) of tumors in c were statistically analyzed (E). The body weights of nude mice (g) during the experiment were graphically depicted (F).

It has been reported that overexpression of RAD51 increases the resistance of NSCLC patients to platinum agents, leading to a worse prognosis [19, 20]. Moreover, an increasing number of researchers have suggested that targeting RAD51 using specific antisense oligonucleotides or specific chemotherapeutic drugs or antibodies was a promising strategy to help lung cancer patients to reduce the chance for acquiring resistance to gefitinib, cisplatin, mitomycin C (MMC) and other therapeutic agents [21]. Taken above data into consideration, we further investigated the possibility of RAD51 inhibition in increasing the chemosensitivity against (−)-Guaiol in NSCLC cells. Firstly, data from flow cytometry analysis showed that RAD51 inhibition significantly increased the sensitivity of A549 cells to (−)-Guaiol, illustrated by the obvious elevation of cell apoptosis. Furthermore, the increased sensitivity could be reversed by the use of 3MA (Figure 7A), suggesting that autophagy inhibition would compromise the above chemosensitivity. Additionally, data from clonogenic survival assays using A549 cells stably downregualted RAD51 using shRNAs (Figure 7B) or transiently deprived RAD51 using siRNAs (Supplementary Figure S5) showed that knockdown of RAD51 obviously increased the chemosensitivity of A549 cells to (−)-Guaiol *in vitro*.

Subsequently, the *in vivo* chemosensitivity assays using tumor-bearing nude mice were performed (Figure 7C). The tumor growth curves indicated by the tumor volumes (Figure 7D) and the tumor weights (Figure 7E) both showed that A549/shRAD51 #1 cells were more sensitive to (−)-Guaiol, consolidating that RAD51 inhibition would greatly enhance the chemosensitivity of NSCLC cells to (−)-Guaiol. Excitingly, consistent with data in Figure 2D, there was no statistical significance between the body weights of nude mice in (−)-Guaiol treated group and those in NaCl treated group (Figure 7F). All in all, in line with previous reports, our data suggest that RAD51 inhibition is a promising strategy to help reduce the chemoresistance of (−)-Guaiol in NSCLC cells.

### RAD51 is highly overexpressed in lung adenocarcinoma tissues

**Figure 8:**
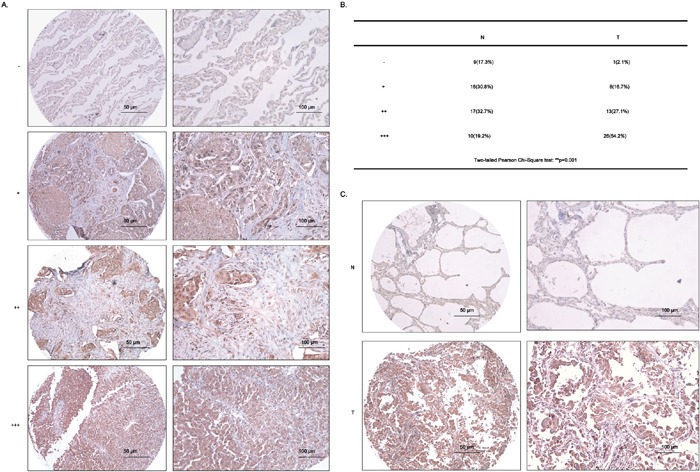
RAD51 is highly upregulated in lung adenocarcinoma tissues **A.** The criteria for the judgement of IHC results from tissue microarray (TMA). **B.** The two-tailed Pearson Chi-Square test of data from IHC assays. N, normal, T, tumor. **C.** Representative data of IHC results from TMA. Scale bars, 50 μm and 100 μm.

RAD51 is commonly recognized as an oncogenic driver in a band of tumors [22] and is highly overexpressed in tumor tissues in patients with poor prognosis and low survival rate [19]. Moreover, the positivity of RAD51 was closely related to squamous carcinoma and poor differentiation in NSCLC patients [20]. Therefore, next, we detected the expression of RAD51 in 100 cases of clinical samples, composed of 52 cases of normal lung tissues (N) and 48 cases of lung adenocarcinoma tissues (T), using tissue microarray (TMA), the detailed information of which was summarized in Supplementary Table S1. According to the criteria scoring RAD51 expression into four grades depending on the intensity and percentage of its staining in NSCLC cells (Figure 8A), the statistical data from Pearson Chi-square tests showed that the expression of RAD51 was dramatically overexpressed in the T tissues (54.2% for +++), in comparison with N tissues (only 19.2% for +++) (Figure 8B). The representative IHC data were shown in Figure 8C. Surprisingly, there is no statistical difference between RAD51 expression in T tissues and the clinical characteristics, including age, gender, tumor grade, tumor stage, tumor TNM stage and lymphatic metastasis (Supplementary Table S2).

## DISCUSSION

At present, the most attractive strategies for the treatment of NSCLC in clinical trials are the oncogenic-molecular targeted chemotherapies [23]. The molecular subtypes of these oncogenic drivers which cause aberrant cellular proliferation, growth and survival include *EGFR, KRAS, HER2, MYC, MET, EML4-ALK*, *BCL2*, *BRAF*, *ILGF-1*, PI3K, DDR2, IGF-1R, FGFR, NTRK1 [24]. Moreover, in accordance with 2014 NCCN clinical practice guidelines in oncology (NCCN guidelines) for NSCLC, the analysis of mutations for EGFR and ALK is widely recommended in clinic [25]. Unfortunately, NSCLC patients usually acquire the resistance to these molecularly targeted drugs within 1-2 years by generating novel mutations that can prevent target inhibition (“gatekeeper” mutations) or activating compensatory signaling pathways (“bypass track” mutations) [7]. As a result, it is imperative to discover new chemotherapeutic drugs with higher efficiency in tumor inhibition and less side-effects. In the current study, we discovered that (−)-Guaiol, a component of a majority of medicinal plants, especially Chinese traditional herbs, significantly inhibited the growth of NSCLC cells both *in vitro* and *in vivo*. The mechanistic studies demonstrated that it targeted RAD51 for lysosome degradation to induce DSBs-triggered cell apoptosis, possibly via caspase signalings. Moreover, downregulation of RAD51 could obviously enhance the sensitivity of NSCLC cells to (−)-Guaiol, which could be partially impaired by autophagy inhibition. Consequently, our results exhibit a new mechanism that is responsible for the tumor therapy and the reduction of the acquired resistance in patients with NSCLC.

From the *in vivo* tumor chemotherapy assays, although there were no statistical difference in tumor volumes and tumor weights between (−)-Guaiol and cisplatin treated groups at the same concentration 8 mg/kg, the body weights of nude mice treated with cisplatin, rather than (−)-Guaiol, were significantly reduced when compared with NaCl group. These data implicated that (−)-Guaiol was likely to have less side-effects in clinical trials than cisplatin. Then the high throughput analysis of the potential targeted genes in (−)-Guaiol treated A549 cells identified 391 downregulated genes which were possibly involved in regulation of cell cycle, DNA replication, DNA binding and chromosome stability. Taking these into consideration, the potential target RAD51 from these downregulated genes was selected to be further studied.

Interestingly, results from western blotting assays, IHC and confocal IF assays confirmed that (−)-Guaiol significantly reduced RAD51 expression to generate detrimental DSBs, leading to cell apoptosis. These data thus prompted us to further investigate how (−)-Guaiol regulated RAD51 protein level. In mammalian cells, the ubiquitination-proteasome pathway and autophagy-lysosome pathway are the two main ways in regulating protein stability [26, 27]. Therefore, in the study, the appropriate inhibitors of these two ways were put into practice to identify the underlying mechanisms for the downregulation of RAD51 in (−)-Guaiol treated cells. Interestingly, expression of RAD51 was specifically rescued by autophagy inhibitor 3MA, not by the proteasome inhibitor MG132, suggesting that (−)-Guaiol targeted RAD51 for the lysosome-mediated protein degradation. However, the detailed molecular mechanisms by which (−)-Guaiol regulates RAD51 expression via autophagy remain to be investigated in the further studies.

It is well-known that RAD51 is a pivotal regulator in homologous recombination repair which is one of the essential repair ways for lethal DSBs in mammalian cells [28]. Consequently, it is rational to think that downregulation of RAD51 will lead to the increase of DSBs in cells. In line with our hypothesis, 3MA, which restored the expression of RAD51 in (−)-Guaiol treated NSCLC cells, significantly inhibited the elevation of DSBs, which eventually activate cell apoptosis to eliminate dead cells [4]. Consistently, further data from flow cytometry assays confirmed the reduction of cell apoptosis in 3MA and (−)-Guaiol treated cells. Notably, the increased apoptotic cells in (−)-Guaiol treated cells were not completely inhibited by 3MA, implying that there were other factors mediating its antitumor activity. It is well known that the caspase activities play a pivotal role in cell apoptosis [29, 30]. Consequently, we investigated whether the DSBs-triggered cell apoptosis in A549 cells was attributed to the increase of caspase activities. Surprisingly, data from flow cytometry assays showed that the cell apoptosis was greatly reduced in Z-VAD and (−)-Guaiol treated cells, in comparison with (−)-Guaiol treated cells, to the same extent as 3MA, implicating that (−)-Guaiol targeted RAD51 to the lysosome degradation, leading to the DSBs-triggered cell apoptosis via caspase activities. However, further studies on the specific caspase enzymes activated by (−)-Guaiol are still required.

Downregulation of RAD51 is beneficial for the enhancement of chemosensitivity to cisplatin, MMC [31], gefitinib, IressaR [27], emodin [32]. Consistently, inhibition of RAD51 significantly increased the sensitivity of A549 cells to (−)-Guaiol which supplies an alternative application of (−)-Guaiol in combination with RAD51 targeted small molecules in NSCLC treatments. Afterwards, we detected the expression of RAD51 in lung adenocarcinoma tissues using IHC assays and statistically analyzed the correlation of RAD51 with clinical properties. Surprisingly, although RAD51 was highly overexpressed in the adenocarcinoma tissues, its overexpression was not related with any clinical characteristics. Studies from other researchers have confirmed that high-level expression of RAD51 has been shown to be related with median survival time, tumor differentiation, clinical stage, squamous pathology and lymphatic metastasis [19]. We consider that this inconsistent result is attributed to the insufficient clinical properties in our present study, further investigations are still needed.

Given the significant role of (−)-Guaiol in inhibiting NSCLC cell growth, there is still a long way in putting (−)-Guaiol into clinical practice. Some issues are raised in the study, for instance, whether it helps NSCLC patients to overcome the acquired resistance in present molecularly targeted drugs, whether (−)-Guaiol alone or together with other therapeutic drugs improves the overall survival ratio of NSCLC patients, whether there are side-effects like acquired resistance caused by (−)-Guaiol. More studies are required to clear these obstacles hindering on the road of applying (−)-Guaiol to benefit NSCLC patients in clinic. In summary, our studies not only provide a novel chemotherapeutic candidate with the same efficiency in tumor inhibition as cisplatin but with less side-effects, but also help to develop an attractive strategy in designing the therapeutic combination to increase the chemosensitivity of NSCLC patients.

## MATERIALS AND METHODS

### Cell culture and drug administration

The NSCLC cell lines (A549, H1299) purchased from American Type Culture Collection (ATCC) and the normal lung cells BEAS-2B, commercially obtained from the FuDan IBS cell center, were all maintained in complete Dulbecco's Modified Eagle' Medium (DMEM) (Gibco), supplemented with 10% FBS (Gibco), 100 units/ml penicillin and 100 μg/ml streptomycin, at 37°C in a humidified incubator with 5% CO_2_. The phenotype of cells used in the study were shown in Supplementary Figure S6. (−)-Guaiol, commercially obtained from Sigma, was diluted in methanol at a primary stock concentration 20 mM. 3MA, purchased from Sigma, was diluted in phosphate buffer saline (PBS, pH=8.0) at a stock concentration 0.5 M. MG132, a proteasome inhibitor [33], was diluted in DMSO at a final concentration 40 mM. The pan-caspase inhibitor Z-VAD-FMK (Z-VAD) was obtained from Selleck and diluted in DMSO at a stock concentration 5 mM. For the treatments, cells were treated with 150 μM (−)-Guaiol for 24 h in serum free medium, unless otherwise indicated. For the inhibition of autophagy or proteasome, 5 mM 3MA in serum free medium or 40 μM MG132 in complete medium were used after (−)-Guaiol treatment for another 6 h. For the caspase activity inhibition, 20 μM Z-VAD was incubated with (−)-Guaiol in serum free medium for 24 h.

### Plasmids construction and cell transfection

The full length of human RAD51 was amplified and ligated into the pEGFP-C3 vector (GFP) between Sal I and BamH I sites, denoted as GFP-RAD51. The siRNAs against non-specific sequence (siCon.) or different sequences of RAD51 (siRAD51 #1, #2) were purchased from Sigma. And the short hairpin RNAs (shRNA) targeting non-specific sequence (shCon.) or different sequences of RAD51 (shRAD51 #1, #2) were amplified and inserted into pLKO.1 vector (Addgene). Transfection of siRNAs or plasmids and establishment of stable cell lines were all performed according to our previous experimental procedures [34]. Primers of plasmids, siRNAs and shRNAs used in this work were summarized in Supplementary Table S3.

### Antitumor activity analysis *in vitro* and *in vivo*

To investigate the potential roles of (−)-Guaiol in tumor inhibition, the *in vitro* colony formation assays and cell counting kit-8 (CCK8) assays were performed and the *in vivo* chemotherapeutic tumor-bearing nude mice assays were also carried out according to our previous protocols [34–36]. IC_50_ values of (−)-Guaiol on NSCLC cells and normal lung cells were evaluated using SPSS statistics. Notably, in the xenograft experiments, 8 mg/kg (−)-Guaiol was intraperitoneally injected three times per week, taking NaCl as a negative control and the cisplatin treatment (8 mg/kg, Sigma) as a positive control.

### High throughput gene expression assay

For the high throughput gene expression assay, total RNA extracted from A549 cells after treatments was quantified by the NanoDrop ND-2000 (Thermo Scientific) and its integrity was assessed using Agilent Bioanalyzer 2100 (Agilent Technologies). Then the RNA was transcribed into cDNA, which was subsequently labeled with biotin and hybridized onto the Affymetrix Human PrimeView microarray. After washing and staining, the arrays were scanned by the Affymetrix Scanner 3000. Afterwards, the Affymetrix GeneChip Command Console (Version 4.0) was used to analyze array images to get raw data. Next, the Genespring software (Version 13.1, Agilent Technologies) was further used to achieve the basic analysis of the raw data. Differentially expressed genes were identified through fold change. The threshold set for up- and down-regulated genes was a fold change ≥2.0. Finally, the GO analysis including biological process, molecular function, cellular component and KEGG was performed to determine the possible roles of these differentially expressed genes using online PANTHER. Taking the -Log10 (*p*Value) as the X-axis, the top five categories in GO analysis were shown.

### Autophagic flux assay

The autophagic lentiviruses expressing Stub-RFP-Sens-GFP-LC3 wild type (GFP-RFP-LC3 WT) or mutant type (GFP-RFP-LC3 Mut) deficiency of glycine at 120 site were commercially purchased from GENECHEM (Shanghai, China). According to the manufacture's protocol, A549 cells were infected with the lentiviruses for 36 h and then selected with 2 μg/ml puromycin (Sigma) for 72 h. Finally, the efficiency was confirmed by observing the expression of GFP and RFP under the inverted fluorescence microscope (Leica). Then, the stable A549 GFP-RFP-LC3 WT or Mut cells were seeded into the 24-well plates with coverslips and treated with or without (−)-Guaiol the next day. Afterwards, the cells on the coverslips were fixed with 4% paraformaldehyde (PFA), perforated with 0.5% Triton X-100, and stained with 4′,6-diamidino-2-phenylindole (DAPI) for 2 min at room temperature away from light. Finally, the slides were sealed and pictured under the inverted confocal fluorescence microscope (Zeiss).

### *In vivo* chemosensitivity analysis

According to our previous protocol [34], the male nude mice aged 4 weeks were subcutaneously injected with A549/shCon. cells (600 × 10^4^ cells) on the left side and A549/shRAD51 #1 (600 × 10^4^ cells) on the right side, respectively. When the tumor volumes were about 200 mm^3^, NaCl or 8 mg/kg (−)-Guaiol was intraperitoneally injected at the indicated days. The tumor sizes and mice body weights were recorded during the experiment. 30 days after the first drug injection, the mice were kindly put to death and the tumors were dissected in the SPF animal house of Shanghai Tenth People's Hospital of Tongji University, approved by the Animal Experiment Management Committee of Shanghai. The tumor weights were finally recorded.

### Quantitative PCR (qPCR), immunostaining and immunohistochemistry assays

The experimental procedures for qPCR, immunostaining and immunohistochemistry (IHC) assays in this research were all performed according to our previous reports [34, 35]. Additionally, the antibodies and primers used in the study were summarized in Supplementary Table S4 and Supplementary Table S5, respectively. By the way, the TMA used in the IHC study of RAD51 expression in NSCLC clinical samples was commercially purchased from Alenabio Ltd (LC10013a, Xi'an, China).

### Statistical analysis

All the data in the study were represented as means ± standard deviation (STD). The two-tailed Student's *t*-test or ANOVA test were appropriately put into practice. And the Pearson Chi-square test was used to analyze the results from IHC assays. *p<0.05 and **p<0.01 were used to determine the statistical significance.

## SUPPLEMENTARY FIGURES AND TABLES




