# Supplementary material for: (−)-Guaiol regulates RAD51 stability via autophagy to induce cell apoptosis in non-small cell lung cancer
Source: Oncotarget. 2016 Aug 23;7(38):62585–97. doi: 10.18632/oncotarget.11540 (PMC5308748; doi:10.18632/oncotarget.11540)
Supplement: Supplementary file 1 [file oncotarget-07-62585-s001.pdf]

(-)-Guaiol regulates RAD51 stability via autophagy to induce cell apoptosis in non-small cell lung cancer

SUPPLEMENTARY FIGURES AND TABLES

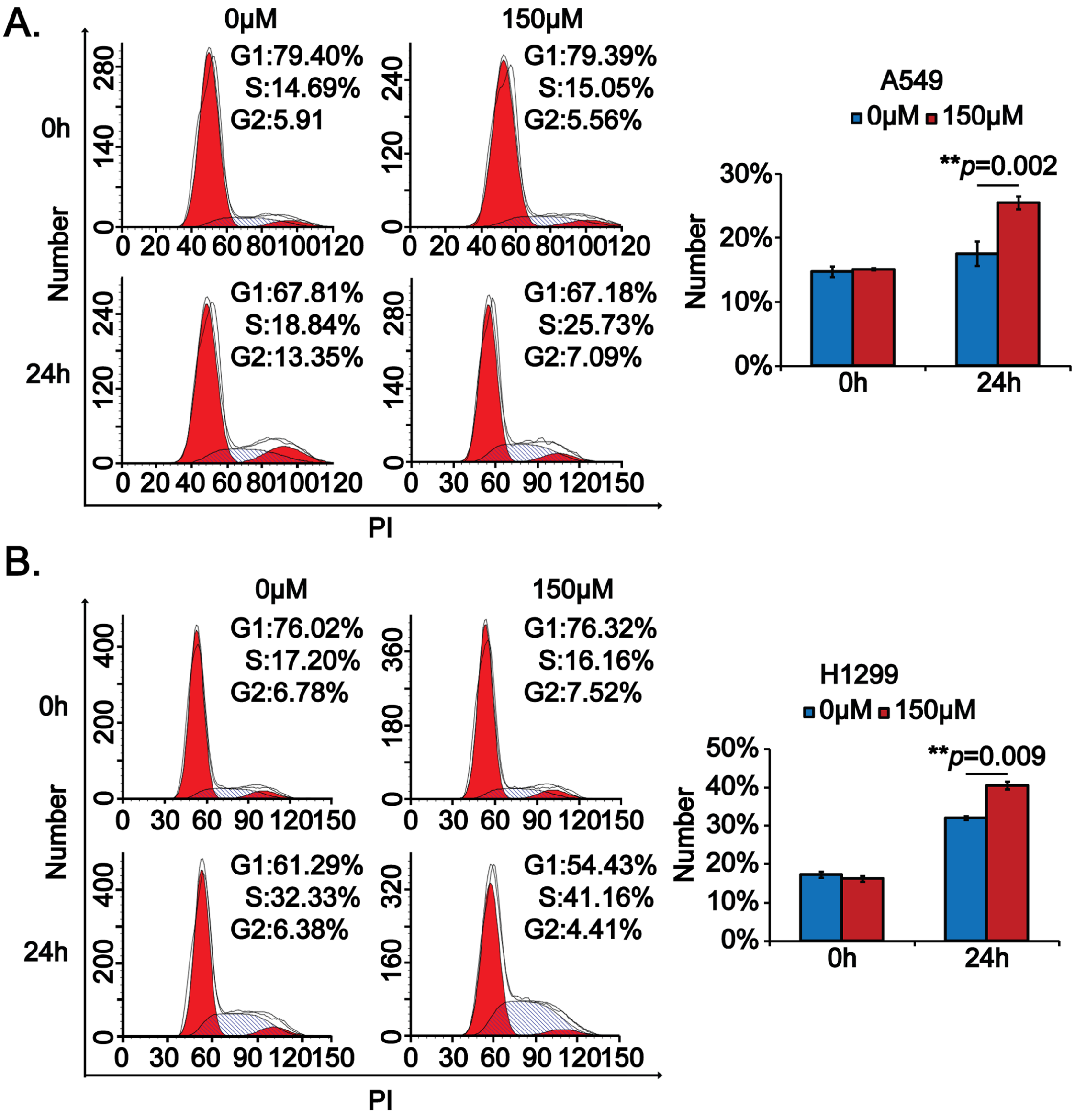

Supplementary Figure S1: (-)-Guaiol induces S phase arrest in NSCLC cells. A-B. Cell cycle analysis of A549 (A) or H1299 (B), starved for 12 h and then recovered for 0 h and 24 h, using flow cytometry assays. Data from three independent experiments were represented as means ± STD and statistically analyzed using Student's *t*-test.

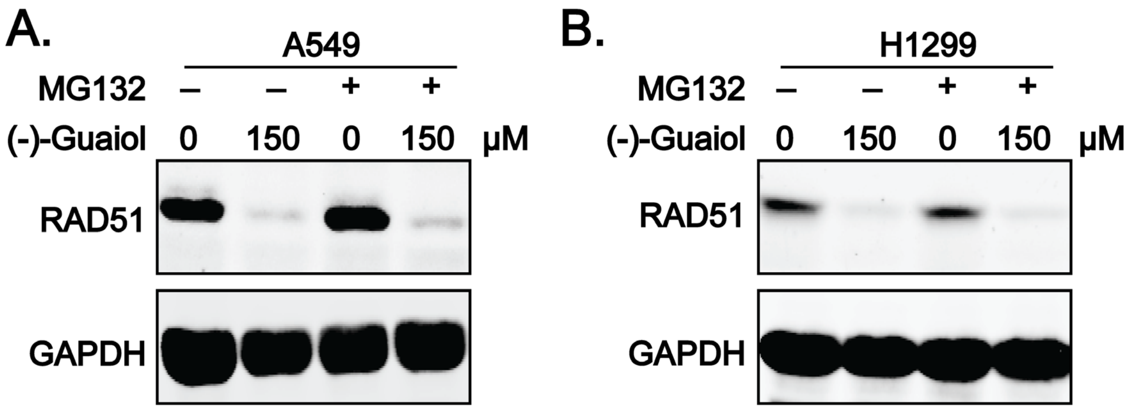

Supplementary Figure S2: (-)-Guaiol does not regulate RAD51 expression via proteasome pathway. A-B. Immunostaining of total protein fromA549 (A) or H1299 (B) cells, treated with or without (-)-Guaiol and further incubated with MG132, with indicated antibodies.

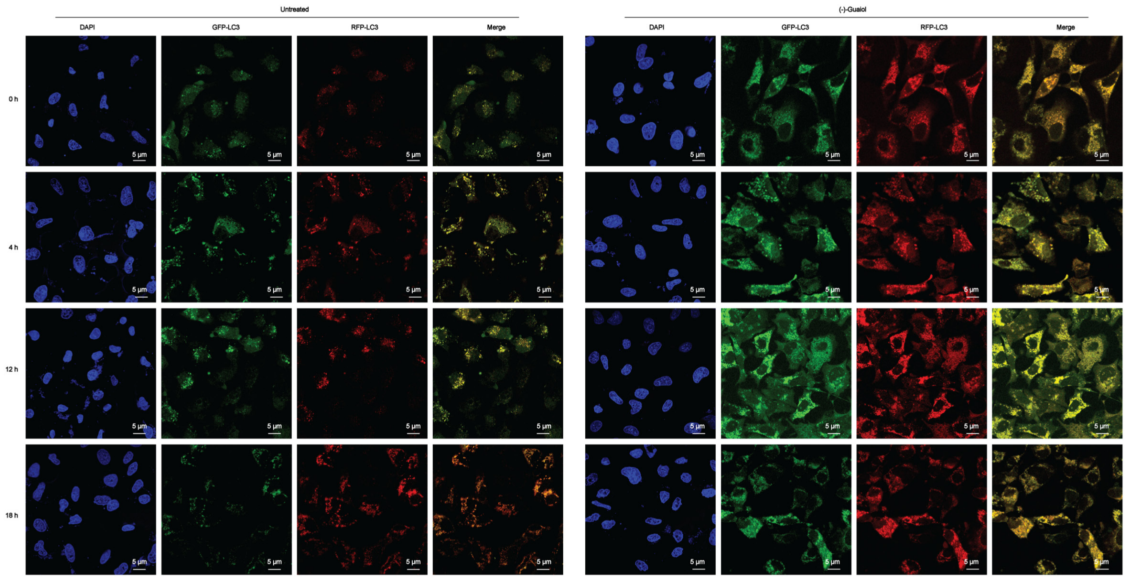

Supplementary Figure S3: Dynamic analysis of autophagic flux in A549 cells treated with or without (-)-Guaiol and then recovered with fresh medium for the indicated time points.

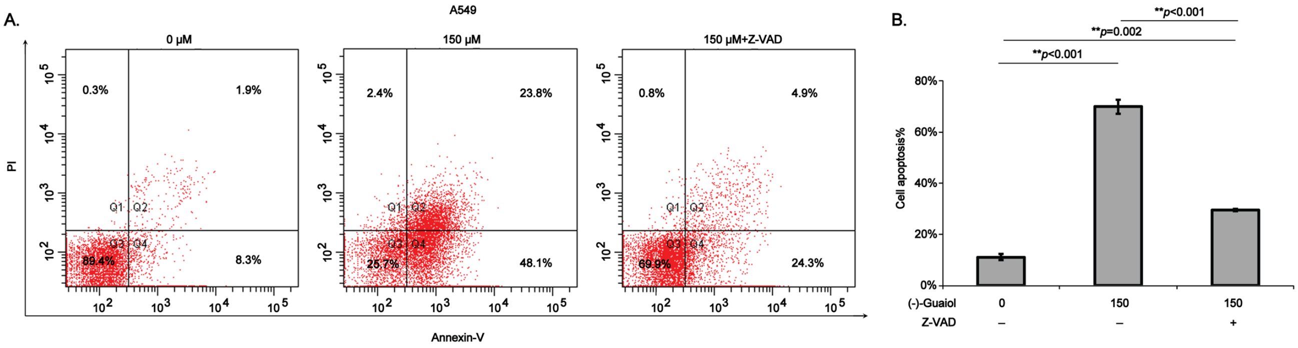

Supplementary Figure S4: (-)-Guaiol induces cell apoptosis via caspase activity. A-B. Cell apoptosis analysis of A549 cells treated with or without (-)-Guaiol or (-)-Guaiol together with pan-caspase inhibitor V-ZAD-FMK (V-ZAD) (A). Data from three independent experiments were statistically analyzed (B). Error bars, STD.

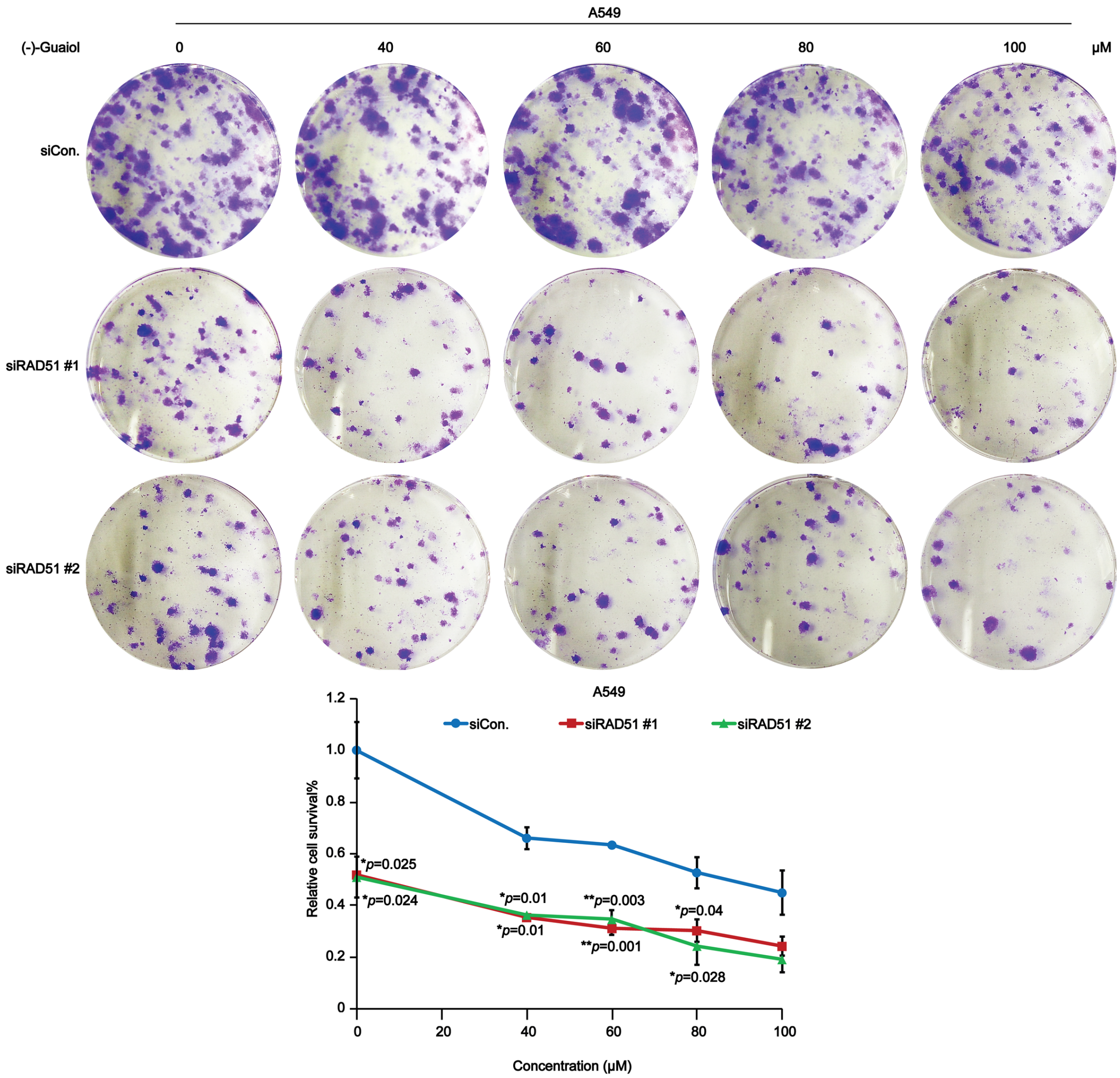

Supplementary Figure S5: RAD51increases resistance of A549 cells to (-)-Guaiol *in vitro*.

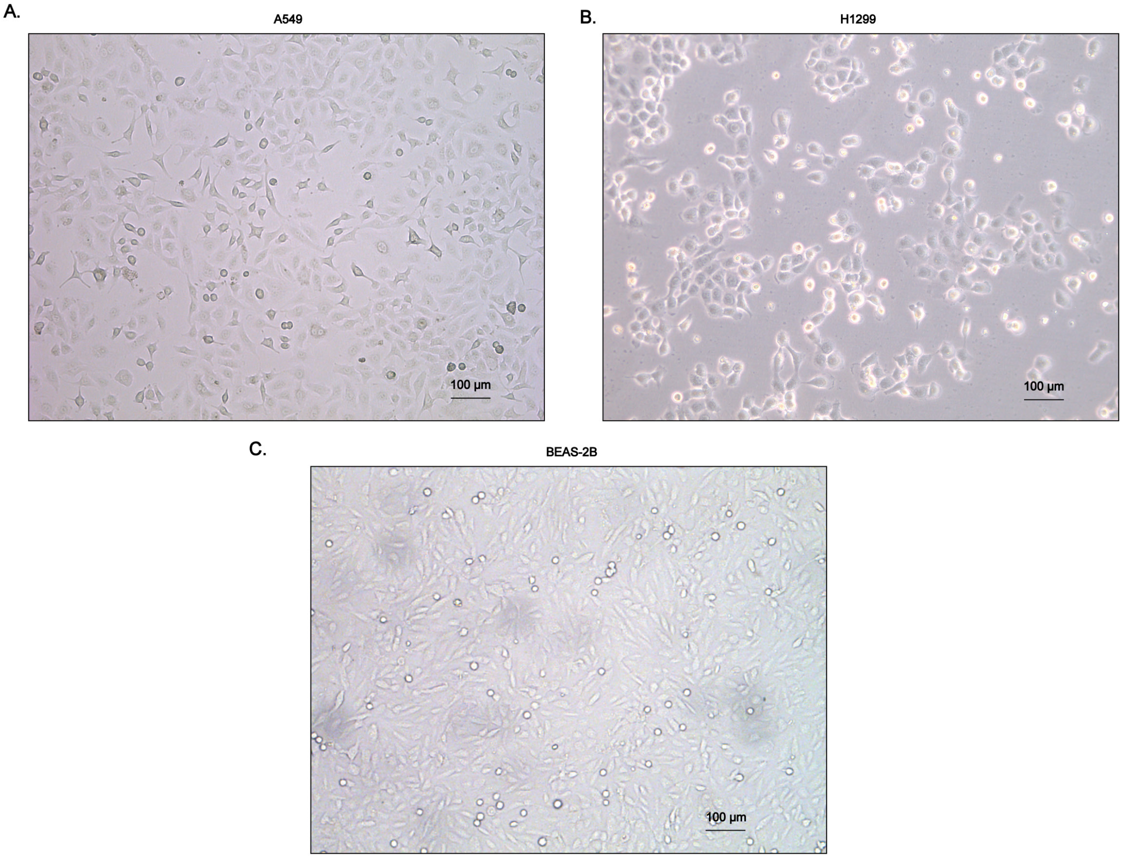

Supplementary Figure S6: Phenotype of NSCLC cells and normal lung cells used in the study. A-C. Phenotypes of A549 (A) or H1299 (B) or BEAS-2B (C) taken under the inverted microscope. Scale bar, μm.

Supplementary Table S1: Clinical characteristics of NSCLC samples in the lung TMA

See Supplementary File 1

Supplementary Table S2: Chi-square test of relationship between RAD51 expression and clinical properties in lung adenocarcinoma tissues

	Total number	Lung adenocarcinoma tissues				Chi-square test
		-	+	++	+++	
**Age**						
**<60**	27	0	6	9	12	
**>=60**	21	1	2	4	14	0.222
**Gender**						
**Male**	24	1	4	5	14	
**Female**	24	0	4	8	12	0.605
**Grade**						
**1**	0	0	0	0	0	
**2**	22	0	3	6	13	
**3**	25	0	5	7	13	0.824
**Stage**						
**I**	29	0	5	8	16	
**II**	11	1	2	2	6	
**III**	7	0	1	2	4	
**IV**	1	0	0	1	0	0.697
**TNM**						
**1**	3	0	0	1	2	
**2**	38	1	7	11	19	
**3**	6	0	1	1	4	
**4**	1	0	0	0	1	0.984
**Lymphatic metastasis**						
**-**	34	0	6	9	19	
+	14	1	2	4	7	0.462

Supplementary Table S3: Primers of siRNAs and plasmids used in the study

Name	Forward/Sense primer	Reverse/Antisense primer
siCon.	UUCUCCGAACGUGUCACGUTT	ACGUGACACGUUCGGAGAATT
siRAD51 #1	CUAAUCAGGUGGUAGCUCAdTdT	UGAGCUACCACCUGAUUAGdTdT
siRAD51 #2	CUGUCUACUGGACAAUCUUdTdT	AAGAUUGUCCAGUAGACAGdTdT
shRAD51 #1	CCGGAACTAATCAGGTGGTAGCTCACTCGA GTGAGCTACCACCTGATTAGTTTTTTG	AATTCAAAAAAACTAATCAGGTGGTAGCTCA CTCGAGTGAGCTACCACCTGATTAGTT
shRAD51 #2	CCGGAACTGTCTACTGGACAATCTTCTCGAG AAGATTGTCCAGTAGACAGTTTTTTG	AATTCAAAAAAACTGTCTACTGGACAATCTT CTCGAGAAGATTGTCCAGTAGACAGTT
GFP-RAD51	TAAGTCGACG ATGGCAATGCAGATGCAGC	CACGGATCCTCAGTCTTTGGCATCTCCCACT

Supplementary Table S4: Antibodies used in the study

Antibody name	Brand	Category NO.	Dilution
GAPDH	SungeneBiotech	KM9002	1:10000
β-actin	Sigma	A1978	1:10000
γH2AX	Abcam	ab26350	1:1000
LC3	Cell signaling technology	#4108	1:1000
RAD51	Abcam	ab88572	1:500
GFP	Santa	sc-8334	1:500
IRDye 800CW goat anti-mouse IgG(H+L)	Licor	926-32210	1:2000
IRDye 800CW goat anti-rabbit IgG(H+L)	Licor	926-32211	1:2000
Anti-mouse IgG (H+L), F(ab')2 Fragment (Alexa Fluor®488 Conjugate)	Cell signaling technology	#4408	1:1000

Supplementary Table S5: Primers of selected differentially expressed genes used in qPCR analysis

	Expression	Former primer	Reverse primer	Length(bp)
18S	NC	CCTGGATACCGCAGCTAGGA	GCGGCGCAATACGAATGCCCC	112
BMP2	NC	GCGGTCTCCTAAAGGTCG	TCAGAGGGCTGGGATGAG	161
Caspase 3	NC	AGAACTGGACTGTGGCATTG	AAGCGACTGGATGAACCAGG	160
P53	NC	GTGAGCGCTTCGAGATGTTC	GCCCTTCTGTCTTGAACATG	165
P21	NC	GACCTGTCACTGTCTTGTAC	GGTAGAAATCTGTCATGCTG	121
UHRF1	-2.572053	CACAACGTGTGCAAGGACTG	TTTCAGCAAAACGCCTGTCG	197
CDK1	-2.1886888	ACTACAGGTCAAGTGGTAGC	AGCACATCCTGAAGACTGAC	137
MAP2K6	-2.702602	AACTCCACTTGCATGAAGATTG	TTTCGCTTCTTGCCTTTCG	149
TP63	-2.5370526	CACTCCTACAACCATTCCTGAT	ACCTCGCTAAGAAACTGACAAT	197
MAP3K8	-2.177209	CTCCGAGGAACAGAGATTTACAT	GGTGCTTGCTTGTGGATTATG	185
BRIP1	-2.1215477	GCAACAATCTCTTAGTGGGAAAC	GGTGGTGTGCTTGGATAGT	156
FANCD2	-2.2691789	ATGCTCTGCTCTGTATCTACCT	AGCCATCATCACACGGAAG	173
RAD51	-2.391215	AGTGATGTCCTGGATAATGT	ACCCGAGTAGTCTGTTCTGT	156
PARP1	-2.029085	ATCGAGGTGGCCTACAGTCT	CGCCTTCACGCTCTATCTTA	220
IRAK2	2.4665444	CCAGGCAACCGATGACTT	GGGTGGCAGCATCTAAGAC	189
EGFR	2.3479931	GCCGCAAAGTGTGTAACG	ATCCAGAGGAGGAGTATGTGT	167
RBCK1	2.2929146	ACCTGCCCTGTGTGTTTC	GCATCACCTTCAGCATCTCT	286
IL6	2.1906278	GTGAGGAACAAGCCAGAG	GCTACATTTGCCGAAGAG	234

NC, normal control.
