# Supplementary material for: (−)-Guaiol regulates RAD51 stability via autophagy to induce cell apoptosis in non-small cell lung cancer
Source: Oncotarget. 2016 Aug 23;7(38):62585–97. doi: 10.18632/oncotarget.11540 (PMC5308748; doi:10.18632/oncotarget.11540)
Supplement: Supplementary file 2 [file oncotarget-07-62585-s002.docx]

**Table S1. Clinical characteristics of NSCLC samples in the lung TMA.**

| **Position** | **Gender** | **Age** | **Pathology** | **Grade** | **Stage** | **TNM** | **Type** | **RAD51 expression** |
| --- | --- | --- | --- | --- | --- | --- | --- | --- |
| A1 | F | 42 | Adenocarcinoma | 2 | IIIB | T4N1M0 | Malignant | **+++** |
| A2 | F | 39 | Adenocarcinoma | 2 | I | T2N0M0 | Malignant | **++** |
| A3 | M | 50 | Adenocarcinoma | 3 | IIB | T3N0M0 | Malignant | **+++** |
| A4 | M | 75 | Adenocarcinoma with necrosis | 2 | I | T2N0M0 | Malignant | **+** |
| A5 | F | 59 | Adenocarcinoma | 2 | IIB | T2N1M0 | Malignant | **+** |
| A6 | M | 62 | Adenocarcinoma | 2 | IV | T2N0M1 | Malignant | **++** |
| A7 | M | 51 | Adenocarcinoma | 2 | IIA | T2N1M0 | Malignant | **++** |
| A8 | M | 49 | Adenocarcinoma | 3 | II | T1N1M0 | Malignant | **++** |
| A9 | M | 59 | Adenocarcinoma | 2 | II | Y2N1M0 | Malignant | **+++** |
| A10 | M | 65 | Adenocarcinoma | 2 | IIIA | T3N2M0 | Malignant | **+++** |
| B1 | F | 54 | Adenocarcinoma | 3 | IIIA | T3N1M0 | Malignant | **++** |
| B2 | M | 61 | Adenocarcinoma | - | II | T2N1M0 | Malignant | **-** |
| B3 | F | 59 | Adenocarcinoma with necrosis | 2 | I | T2N0M0 | Malignant | **++** |
| B4 | F | 65 | Adenocarcinoma | 2 | I | T2N0M0 | Malignant | **+++** |
| B5 | M | 51 | Adenocarcinoma | 2 | I | T1N0M0 | Malignant | **+++** |
| B6 | F | 37 | Adenocarcinoma | 2 | I | T2N0M0 | Malignant | **++** |
| B7 | F | 52 | Adenocarcinoma | 2 | II | T2N1M0 | Malignant | **+++** |
| B8 | F | 52 | Adenocarcinoma | 2 | II | T2N1M0 | Malignant | **+++** |
| B9 | M | 62 | Adenocarcinoma | 2 | I | T2N0M0 | Malignant | **+++** |
| B10 | F | 49 | Adenocarcinoma | 2 | I | T2N0M0 | Malignant | **+++** |
| C1 | F | 42 | Adenocarcinoma | 2 | I | T2N0M0 | Malignant | **+++** |
| C2 | M | 55 | Adenocarcinoma | 2 | I | T2N0M0 | Malignant | **+++** |
| C3 | M | 70 | Adenocarcinoma with necrosis | 2 | I | T2N0M0 | Malignant | **++** |
| C4 | M | 52 | Adenocarcinoma | 3 | II | T2N1M0 | Malignant | **+** |
| C5 | F | 37 | Adenocarcinoma | 3 | I | T2N0M0 | Malignant | **+** |
| C6 | F | 63 | Adenocarcinoma | 2 | I | T2N0M0 | Malignant | **+** |
| C7 | F | 64 | Adenocarcinoma | 3 | I | T2N0M0 | Malignant | **++** |
| C8 | M | 70 | Adenocarcinoma | 3 | I | T2N0M0 | Malignant | **+++** |
| C9 | M | 40 | Adenocarcinoma | 3 | I | T2N0M0 | Malignant | **+** |
| C10 | F | 62 | Adenocarcinoma | 3 | I | T2N0M0 | Malignant | **+++** |
| D1 | M | 64 | Adenocarcinoma | 3 | I | T2N0M0 | Malignant | **++** |
| D2 | M | 63 | Adenocarcinoma | 3 | I | T2N0M0 | Malignant | **+++** |
| D3 | M | 58 | Adenocarcinoma | 2 | I | T2N0M0 | Malignant | **+++** |
| D4 | F | 58 | Adenocarcinoma | 3 | I | T2N0M0 | Malignant | **++** |
| D5 | F | 32 | Adenocarcinoma | 3 | I | T2N0M0 | Malignant | **++** |
| D6 | M | 69 | Adenocarcinoma with necrosis | 3 | I | T2N0M0 | Malignant | **+++** |
| D7 | F | 68 | Adenocarcinoma (sparse) | 3 | II | T2N1M0 | Malignant | **+++** |
| D8 | F | 61 | Adenocarcinoma | 3 | I | T2N0M0 | Malignant | **+++** |
| D9 | M | 62 | Adenocarcinoma | 3 | IIIA | T3N0M0 | Malignant | **+++** |
| D10 | F | 72 | Adenocarcinoma | 3 | II | T2N1M0 | Malignant | **+++** |
| E1 | M | 60 | Adenocarcinoma | 3 | I | T2N0M0 | Malignant | **+++** |
| E2 | M | 49 | Adenocarcinoma | 3 | I | T2N0M0 | Malignant | **+++** |
| E3 | F | 46 | Adenocarcinoma | 2 | I | T1N0M0 | Malignant | **+++** |
| E4 | M | 65 | Adenocarcinoma | 3 | IIIA | T3N0M0 | Malignant | **+++** |
| E5 | F | 62 | Adenocarcinoma | 3 | I | T2N0M0 | Malignant | **+++** |
| E6 | F | 36 | Adenocarcinoma | 3 | IIIA | T2N2M0 | Malignant | **++** |
| E7 | M | 39 | Adenocarcinoma | 3 | I | T2N0M0 | Malignant | **+** |
| E8 | F | 58 | Adenocarcinoma | 3 | IIIA | T3N0M0 | Malignant | **+** |
| E9 | F | 42 | Adjacent normal lung tissue | - | - | - | NAT | **-** |
| E10 | F | 39 | Adjacent normal lung tissue | - | - | - | NAT | **++** |
| F1 | M | 50 | Emphysema | - | - | - | NAT | **-** |
| F2 | M | 75 | Adjacent normal lung tissue | - | - | - | NAT | **-** |
| F3 | F | 59 | Adjacent normal lung tissue | - | - | - | NAT | **+** |
| F4 | M | 62 | Adjacent normal lung tissue | - | - | - | NAT | **++** |
| F5 | M | 51 | Adjacent normal lung tissue | - | - | - | NAT | **++** |
| F6 | M | 49 | Adjacent normal lung tissue | - | - | - | NAT | **-** |
| F7 | M | 59 | Adjacent normal lung tissue | - | - | - | NAT | **+** |
| F8 | M | 65 | Pulmonary collapse | - | - | - | NAT | **++** |
| F9 | F | 54 | Pulmonary collapse | - | - | - | NAT | **-** |
| F10 | M | 61 | Adjacent normal lung tissue | - | - | - | NAT | **+** |
| G1 | F | 59 | Adjacent normal lung tissue | - | - | - | NAT | **+++** |
| G2 | F | 65 | Adjacent normal lung tissue | - | - | - | NAT | **+++** |
| G3 | M | 51 | Adjacent normal lung tissue | - | - | - | NAT | **++** |
| G4 | F | 37 | Adjacent normal lung tissue | - | - | - | NAT | **+++** |
| G5 | F | 52 | Adjacent normal lung tissue | - | - | - | NAT | **++** |
| G6 | F | 52 | Adjacent normal lung tissue | - | - | - | NAT | **+++** |
| G7 | M | 62 | Adjacent normal lung tissue | - | - | - | NAT | **+** |
| G8 | F | 49 | Adjacent normal lung tissue | - | - | - | NAT | **++** |
| G9 | F | 42 | Adjacent normal lung tissue | - | - | - | NAT | **++\** |
| G10 | M | 55 | Emphysema | - | - | - | NAT | **+++** |
| H1 | M | 70 | Adjacent normal lung tissue | - | - | - | NAT | **+++** |
| H2 | M | 52 | Adjacent normal lung tissue | - | - | - | NAT | **++** |
| H3 | F | 37 | Adjacent normal lung tissue | - | - | - | NAT | **++** |
| H4 | F | 63 | Adjacent normal lung tissue | - | - | - | NAT | **++** |
| H5 | F | 64 | Adjacent normal lung tissue with tumor embolus | - | - | - | NAT | **+++** |
| H6 | M | 70 | Adjacent normal lung tissue | - | - | - | NAT | **+** |
| H7 | M | 40 | Adjacent normal lung tissue | - | - | - | NAT | **++** |
| H8 | F | 62 | Adjacent normal lung tissue | - | - | - | NAT | **+** |
| H9 | M | 64 | Adjacent normal lung tissue | - | - | - | NAT | **+** |
| H10 | M | 63 | Adjacent normal lung tissue | - | - | - | NAT | **++** |
| I1 | M | 58 | Adjacent normal lung tissue | - | - | - | NAT | **++** |
| I2 | F | 58 | Adjacent normal lung tissue | - | - | - | NAT | **-** |
| I3 | F | 32 | Adjacent normal lung tissue | - | - | - | NAT | **+** |
| I4 | M | 69 | Adjacent normal lung tissue | - | - | - | NAT | **++** |
| I5 | F | 68 | Pneumonia | - | - | - | NAT | **+++** |
| I6 | F | 61 | Emphysema | - | - | - | NAT | **+++** |
| I7 | M | 62 | Adjacent normal lung tissue | - | - | - | NAT | **+** |
| I8 | F | 72 | Adjacent normal lung tissue | - | - | - | NAT | **-** |
| I9 | M | 60 | Adjacent normal lung tissue | - | - | - | NAT | **++** |
| I10 | M | 49 | Adjacent normal lung tissue | - | - | - | NAT | **+** |
| J1 | F | 46 | Adjacent normal lung tissue | - | - | - | NAT | **+** |
| J2 | M | 65 | Adjacent normal lung tissue | - | - | - | NAT | **+** |
| J3 | F | 62 | Adjacent normal lung tissue | - | - | - | NAT | **+** |
| J4 | F | 36 | Adjacent normal lung tissue | - | - | - | NAT | **++** |
| J5 | M | 39 | Adjacent normal lung tissue | - | - | - | NAT | **-** |
| J6 | F | 58 | Adjacent normal lung tissue | - | - | - | NAT | **+++** |
| J7 | M | 24 | Lung tissue | - | - | - | Normal | **+** |
| J8 | M | 48 | Lung tissue | - | - | - | Normal | **+** |
| J9 | F | 15 | Lung tissue | - | - | - | Normal | **-** |
| J10 | M | 47 | Lung tissue | - | - | - | Normal | **+** |
